# Coronary sinus thrombosis: insights from a comprehensive literature review

**DOI:** 10.1186/s12959-025-00707-x

**Published:** 2025-03-14

**Authors:** Chenlong Yi

**Affiliations:** 1 Department of Cardiovascular Surgery, The Affiliated Hospital of Yangzhou University, Yangzhou University, No. 368 Hanjiang Middle Road, Yangzhou, 225001 Jiangsu Province China; 2 Division of Human Genetics and Translational Medicine, Department of Medicine, University of Pennsylvania, Philadelphia, USA

**Keywords:** Coronary sinus thrombosis (CST), Coronary sinus (CS), Persistent left superior Vena Cava (PLSVC), Anticoagulation, Cardiac surgery

## Abstract

Coronary sinus thrombosis (CST) is a rare and severe disease that remains underrecognized in clinical practice. Most documented cases fall within the acute category, with non-specific symptoms, rapid progression, and a high mortality rate. In contrast, some cases follow an insidious course, leading to the formation of partial or incomplete thrombi that may not cause immediate death but can progress to severe complications over time. This review study the pathophysiology, clinical presentations, diagnostic challenges, and the association with persistent left superior vena cava (PLSVC), emphasizing the utility of imaging modalities such as Echocardiography, Computed Tomography, Magnetic Resonance Imaging and Venography. For management, anticoagulation remains the cornerstone of treatment for CST, with surgical thrombectomy reserved for severe or refractory cases. Post-treatment follow-up is highlighted as essential for monitoring recurrence and managing complications. In brief, the aim of this review is to enhance the understanding of CST and improve clinical outcomes through early recognition, tailored interventions, and multidisciplinary care.

## Pathophysiology of coronary sinus thrombosis (CST)

### The anatomy and function of the coronary sinus (CS)

The CS, located in the posterior heart within the atrioventricular groove, is a critical channel that drains approximately 60% of venous return from major cardiac veins—such as the great cardiac vein, oblique vein of the left atrium, posterior vein of the left ventricle, middle cardiac vein, and small cardiac vein—into the right atrium. This drainage ensures efficient clearance of metabolic byproducts and deoxygenated blood, maintaining myocardial circulation and health [1, 2].

The heart’s venous drainage comprises three systems: The first major system is the CS. The second system, the anterior cardiac veins, handles most of the remaining 40% and also drains into the right atrium, though these two systems have distinct entry points, numerous anastomotic connections allow blood to reroute if resistance increases in one path. The third, minor system is the thebesian veins, which drain directly into all chambers and can occasionally carry up to 50% of venous return [1]. The presence of anastomotic connections between the CS and anterior cardiac veins, along with the auxiliary drainage from the thebesian veins, may play a role in minimizing hemodynamic impact during the formation of CST [1]. However, this does not imply that CST presents benignly in all clinical scenarios, as its severity may vary based on other factors.

### Clinical significance of the CS

The CS holds a central role in multiple cardiac procedures, given its unique anatomical and physiological attributes. Anatomically, the CS is an ideal access point for interventions involving the heart’s venous system. This position is pivotal in electrophysiological procedures, such as cardiac resynchronization therapy (CRT) [3], arrhythmia ablation [4] and for deployment of an array of cardiac devices [5, 6]. In addition, the CS’s accessibility makes it a crucial route for retrograde cardioplegia in cardiac surgeries, particularly in cases where antegrade perfusion is compromised [7]. The CS is also used as a potential access route for ventriculoatrial shunt placement, demonstrating its versatility in certain central venous interventions [8]. Additionally, the dilation of CS can serve as a diagnostic clue for conditions like pulmonary hypertension [9] or congenital defects such as TAPVR [10].

### CS changes in specific pathology

The CS is susceptible to various structural anomalies that can lead to significant pathophysiological changes, especially affecting venous return and creating risks for several cardiovascular complications.

Persistent left superior vena cava (PLSVC) is one of the most frequent congenital variations, most cases with PLSVC exhibit CS dilation due to the additional venous load [11]. The increased pressure within the CS may lead to venous stasis, predisposing individuals to thrombosis formation. Furthermore, an enlarged CS can stretch the atrioventricular node or compress the His bundle, causing conduction disturbances and increasing the risk of arrhythmias [11–14].

In CS atresia (CSA), the entry of the CS into the right atrium is narrowed or absent, causing blood to bypass the CS through collateral veins [15]. Another anomaly, unroofed coronary sinus syndrome (URCS), a segment of the CS wall is missing, creating a direct connection between the CS and the left atrium, leading to an abnormal shunt [16]. These anatomical anomalies both increase venous resistance and divert blood flow, resulting in progressive venous congestion, right atrial overload, and often pulmonary hypertension [17]. In cases of URCS, thrombosis can pose an additional risk of embolism within the systemic circulation [18, 19].

CS diverticulum and aneurysmal dilations represent localized outpouchings or general dilation of the CS, which are often asymptomatic but increase the risk of thrombus formation within the CS due to blood stasis. Additionally, aneurysmal dilations may increase the risk of rupture under high-pressure conditions, leading to pericardial effusion or tamponade in severe cases. Coronary sinus dilatation may also cause mechanical compression of the left side of the heart, leading to mitral valve dysfunction [20], potentially reducing cardiac output.

## CST

Within the spectrum of rare disorders afflicting CS, CST stands out as a severe malady [1]. Most documented cases of CST fall within the acute category [1], presenting a clinical scenario laden with complexities. Non-specific clinical manifestations and rapid progression, combined with its overall rarity, make CST a challenging diagnosis with high mortality. Nevertheless, there exist instances where the onset is insidious, leading to the formation of partial or incomplete thrombi which do not result in immediate mortality. This phenomenon can be attributed to the development of robust collateral circulation [21]. Consequently, this classification may remain clinically silent, devoid of overt signs indicating myocardial decompensation. However, it remains imperative to acknowledge the potential emergence of further complications over time. These complications may encompass the progression to complete occlusion, thrombus rupture, and subsequent embolization, ultimately carrying the fatal risk of conditions [1, 14, 22, 23].

### CST formation mechanisms

The mechanism involving CST has been suspected to be similar to thrombosis at other sites. involving Virchow’s triad of endothelial damage, hypercoagulability, and venous stasis [24, 25]. CST can be classified into two types based on cause: invasive, typically following intracardiac procedures, and spontaneous, which is associated with systemic or local factors.

Invasive CST typically results from central venous catheterization [26] or intracardiac procedures [4, 27–29], which can damage the CS endothelium and trigger thrombus formation. During these interventions, mechanical trauma to the inner lining of the CS exposes subendothelial tissues, initiating the coagulation cascade by activating tissue factors and promoting platelet adhesion and aggregation. Moreover, turbulent blood flow caused by endothelial injury further disrupts local hemodynamics, favoring clot formation by increasing shear stress and promoting localized blood stasis [25].

Spontaneous CST develops without prior endothelial injury or external manipulation and is closely associated with conditions involving venous stasis and hypercoagulability. Venous stasis frequently occurs in patients with conditions like atrial fibrillation [30], right heart failure [28, 31], or congenital malformations such as PLSVC and aneurysmal dilations [32–34], which slow blood flow and lead to stagnation within the CS. This stasis limits the natural clearance of clotting factors, facilitating a prothrombotic environment where clot formation becomes more likely. This heightened prothrombotic state can result from systemic diseases like cancer [35, 36], inflammatory [37–39] or autoimmune disorders [40, 41]. Previous literature has reported certain diseases, such as Crohn’s disease [42], Idiopathic pulmonary fibrosis [43], Kawasaki disease [44], Acute lymphoblastic leukemia [45], Antiphospholipid antibody syndrome [40] can contribute to hypercoagulability. Lifestyle factors like cocaine abuse and specific medications can also elevate coagulability, adding to the risk profile [46–49]. Together, these elements illustrate how a combination of blood stasis and hypercoagulable conditions can synergistically elevate the likelihood of spontaneous CST. In certain conditions, CST can be self-limiting and resolve spontaneously. As noted, CST may develop following myocardial infarction (MI) with ventricular wall rupture; however, as the rupture heals, the thrombosis may resolve without intervention [50]. This highlights the potential for transient CST in specific cardiac events.

### CST and its association with PLSVC

In our retrospective analysis, we observed that more than 20% of patients with CST also presented with PLSVC (Table 1). The presence of PLSVC can alter intrinsic venous return, complicate the clinical course of CST, and necessitate specific therapeutic interventions, such as more aggressive anticoagulation therapy or, in rare cases, surgical intervention.

**Table 1:** Case reports and series for CST

	First author	Age	PLSVC	Procedure	Potential Cause	Diagnosed	Intervention	anticoagulation	Outcome
1	Shimbori R [20]	58 Y	Yes	invasive	Cardiac surgery, Right heart failure, PLSVC	Yes	Surgery	N/A	Alive
2	Dabbah S [29]	70 Y	Yes	invasive	Cardiac surgery, RHC	Yes	Anticoagulation	Heparin, Warfarin, Aspirin	Alive
3	Chaithiraphan S [32]	85 Y	Yes	Invasive	Intravenous pacemaker, infection	No	N/A	N/A	Dead
4	Bapat VN [27]	14 Y	Yes	invasive	Cardiac surgery	No	N/A	N/A	Dead
5	Philips JB 3rd [77]	36 D	Yes	Invasive	CVC	No	N/A	N/A	Dead
6	Yeo KK [4]	11 Y	No	invasive	Electrophysiologic ablation	Yes	Surgery + Anticoagulation	Heparin, Warfarin, Aspirin, Clopidogrel	Alive
7	Lim CW [58]	N/A	No	Invasive	CVC, Infection	Yes	Surgery + Anticoagulation	N/A	Alive
8	Frogel JK [23]	36 Y	No	Invasive	Cardiac surgery	Yes	Surgery	N/A	Alive
10	Kranig W [68]	77 Y	No	Invasive	Cardiac surgery	Yes	Surgery	N/A	Alive
9	Huang HL [3]	N/A	No	Invasive	CRT	Yes	Surgery	N/A	Alive
11	Radermecker MA [21]	65 Y	No	Invasive	The Viacor transvenous device	Yes	Surgery	N/A	Alive
12	Milligan G [14]	70 Y	No	invasive	RHC	Yes	Anticoagulation	Apixaban, Aspirin, Clopidogrel	Alive
13	Luckie M [5]	N/A	No	Invasive	Intravenous pacemaker	Yes	Anticoagulation	Warfarin	Alive
14	Hazan MB [55]	53 Y	No	Invasive	Cardiac surgery, RHC, infection	Yes	N/A	N/A	Alive
15	RC Parmar [54]	63 D	No	invasive	Cardiac surgery, infection	No	N/A	N/A	Dead
16	Ford JC [61]	68 Y	No	invasive	RHC	No	N/A	N/A	Dead
17	Suárez-Peñaranda [22]	9 Y	No	Invasive	CVC	No	N/A	N/A	Dead
18	Figuerola M [26]	67 Y	No	Invasive	CVC	No	N/A	N/A	Dead
19	C A Wells [8]	9 Y	No	Invasive	Ventriculoarterial shunt revise	No	N/A	N/A	Dead
20	Guindi MM [28]	50 Y	No	Invasive	Cardiac surgery, Swan-Ganz	No	N/A	N/A	Dead
21	Guindi MM [28]	64 Y	No	Invasive	Transvenous pacemaker, Swan-Ganz	No	N/A	N/A	Dead
22	Urbanová D [78]	N/A	N/A	Invasive	N/A	No	N/A	N/A	Dead
23	Stevenhagen J [67]	68 Y	Yes	spontaneous	PLSVC, CSA	Yes	Surgery + Anticoagulation	Heparin, Warfarin	Alive
24	Wang W [72]	73 Y	Yes	spontaneous	AF, PLSVC, CSA	Yes	Surgery + Anticoagulation	Heparin, Warfarin	Alive
25	Yamaguchi M [62]	31 Y	Yes	spontaneous	Hemangioma, PLSVC, CSA	Yes	Surgery	N/A	Alive
26	Moey MYY [33]	72 Y	Yes	spontaneous	AF, PLSVC, CSA	Yes	Anticoagulation	Warfarin, Rivaroxaban	Alive
27	Ufuk F [17]	74 Y	Yes	spontaneous	PLSVC, CSA	Yes	Anticoagulation	Heparin	Alive
28	Matsui H [34]	82 Y	Yes	spontaneous	AF, PLSVC, CSA	Yes	Anticoagulation	N/A	Alive
29	Ebin E [75]	72 Y	Yes	spontaneous	PLSVC, CSA	Yes	Anticoagulation	N/A	Alive
30	Krishnamohan P [40]	31 Y	No	spontaneous	SLE, Anti-Phospholipid syndrome	Yes	Surgery + Anticoagulation	Heparin, Warfarin	Alive
31	Neri E [30]	79 Y	No	spontaneous	Heart failure, AF	Yes	Surgery + Anticoagulation	N/A	Alive
32	Liu H [31]	23 Y	No	spontaneous	Right heart failure	Yes	Surgery	N/A	Alive
33	Ojukwu O [63]	67 Y	No	spontaneous	Arteriovenous fistula, CSA	Yes	Surgery	N/A	Alive
34	Jones D [37]	50 Y	No	spontaneous	Bacteremia, CVC, RHC	Yes	Surgery	N/A	Alive
35	Wang H [41]	66 D	No	spontaneous	Kawasaki disease	Yes	Anticoagulation	Heparin, bivalirudin, Aspirin, Clopidogrel	Alive
36	Song G [44]	3 M	No	spontaneous	Kawasaki disease	Yes	Anticoagulation	Heparin	Alive
37	López-Pena AM [57]	72 Y	No	spontaneous	Amyloidosis	Yes	Anticoagulation	Heparin	Alive
38	Ibrahim IA [52]	64 Y	No	spontaneous	Heart failure	Yes	Anticoagulation	Rivaroxaban	Alive
39	Berrin LL [49]	38 Y	No	spontaneous	Cocaine	Yes	Anticoagulation	Rivaroxaban	Alive
40	Krishna Gupta [18]	57 Y	No	spontaneous	AF	Yes	Anticoagulation	Heparin, Dabigatran	Alive
41	Floria M [43]	76 Y	No	spontaneous	Idiopathic pulmonary fibrosis	Yes	Anticoagulation	Heparin, Oral anticoagulants	Alive
42	Güvenç TS [79]	50 Y	No	spontaneous	Heart failure, AF	Yes	Anticoagulation	Warfarin	Alive
43	Kachalia A [80]	66 Y	No	spontaneous	Heart failure,	Yes	Anticoagulation	Heparin, Aspirin. Clopidogrel	Alive
44	Hart MA [81]	61 Y	No	spontaneous	AF, Infection	Yes	Anticoagulation	Warfarin	Alive
45	Norman S [82]	62 Y	No	spontaneous	Malignancy	Yes	N/A	N/A	N/A
46	Martin J [42]	27 Y	No	spontaneous	Crohn’s disease	No	N/A	N/A	Dead
47	Kitazawa S [45]	83 Y	No	spontaneous	Acute lymphoblastic leukemia, Chemotherapy	No	N/A	N/A	Dead
48	Ramsaran EK [53]	71 Y	No	spontaneous	Right atrial infraction	No	N/A	N/A	Dead
49	Dryer R [38]	20 Y	No	spontaneous	Acute bacterial endocarditis	No	N/A	N/A	Dead
50	Ross EM [39]	31 Y	No	spontaneous	Aspergillosis	No	N/A	N/A	Dead
51	Salehi M [50]	60 Y	No	spontaneous	Acute myocardial infarction	Yes	None	N/A	Alive
52	Mararenko A [35]	64 Y	No	spontaneous	Malignancy, bacteremia	Yes	None	N/A	N/A

AF: Atrial fibrillation; CRT: Cardiac resynchronization therapy; CSA: Coronary Sinus atresia; CVC: Central venous catheterization; PLSVC: Persistent Left Superior Vena Cava; RHC: Right heart catheterization; SLE: Systemic lupus erythematosus.

PLSVC, formed from the left anterior cardinal vein, typically regresses during fatal development but persists in 0.2-3% of the general population and up to 11% of patients with congenital heart disease [11]. Its clinical significance often depends on its drainage site and any accompanying anomalies. In most cases, PLSVC drains into the right atrium through the CS without causing notable hemodynamic effects [11]. However, CSA may accompany PLSVC, making the PLSVC the primary retrograde drainage pathway for coronary veins unless collateral drainage pathways develop between the CS and heart chambers [33, 51]. Another scenario arises in patients with a single left superior vena cava (absence of the right superior vena cava), where venous return from the head, neck, and upper limbs relies entirely on the PLSVC entering the CS [32].

As previously noted, PLSVC frequently leads to CS dilation, resulting in venous congestion that promotes gradual thrombus formation. This slow progression, along with the development of collateral circulation, often masks symptoms and can delay diagnosis until the thrombosis has reached a more advanced stage. Therefore, identifying PLSVC early in CST patients provides essential insight into the underlying vascular structure, guiding individualized treatment strategies and potentially modifying therapy. Clinicians are encouraged to promptly screen for PLSVC upon diagnosing CST to ensure a comprehensive approach to management.

## Clinical presentations of CST

The clinical presentation of CST often lacks specific symptoms, making diagnosis particularly challenging. Chronic cases can remain clinically silent, with no overt signs of myocardial decompensation and only vague symptoms in patients. Dyspnea is a common symptom [30, 35, 43], likely resulting from venous stasis, potential cardiac dysfunction [29], pulmonary hypertension, or pulmonary embolism [40, 52]. Furthermore, arrhythmias may result from compression of the conduction bundles [11–14]. Chronic CST is often discovered incidentally during imaging tests, reflecting its frequently asymptomatic nature. However, as the disease progresses or acute CS occlusion occurs, chronic CST can transition to acute CST, with a rapid escalation of symptoms.

Acute CST cases typically present with a sudden and severe clinical picture, often mimicking MI. Common symptoms include sharp chest pain, arrhythmias and hemodynamic instability [1, 4, 49, 50]. Rapid occlusion of the CS by CST raises venous pressure, restricts coronary blood flow, and induces ischemia—even when coronary arteries remain unobstructed [53]. This can lead to congestion in the venous and capillary networks, further exacerbating myocardial injury and contributing to transudative pericardial effusion, which heightens the risk of pericardial tamponade [4, 22, 26, 41]. Acute CST can quickly escalate into life-threatening conditions, including malignant arrhythmias, infarction, acute heart failure, and sudden cardiac death [22, 50, 53]. Due to its rapid progression and tendency to go unrecognized, many acute cases are only identified post-mortem [45].

The rarity and the nonspecific symptoms in chronic cases, which often overlap with other cardiovascular conditions, complicate diagnosis, while acute cases carry a high mortality risk. Early awareness and proactive prevention are essential, and CST should be considered as a differential diagnosis, especially in cases of sudden deterioration after invasive cardiac procedures [4, 27, 54].

## Clinical diagnosis of CST

As previously discussed, CST is often detected incidentally during imaging studies performed for unrelated conditions. Consequently, imaging is essential for confirming the diagnosis and evaluating the extent of thrombosis. Common imaging modalities include the following:

Chest X-ray: Due to the location of the CS, it is challenging to visualize directly on chest X-ray. However, chest X-rays can provide indirect information through signs such as CS dilation, abnormal cardiac silhouette, or when identifying the position of guiding catheters [5, 8, 26, 55].

Electrocardiography (ECG): ECG has limited direct value in diagnosing CST. Common findings, such as arrhythmias or axis deviation, are generally nonspecific. In some chronic CST cases, ECG results may remain normal [17], thus offering value primarily during acute episodes or sudden changes. However, typical ischemic changes, such as ST segment alterations, were hard to distinguish from MI due to overlapping features.

Echocardiography: As the first-line imaging modality for evaluating cardiac abnormalities, both transthoracic echocardiography (TTE) and transesophageal echocardiography (TEE) provide valuable information for diagnosing CST. TTE is a non-invasive, widely available, and easy to perform method. In the apical 2-chamber and 4-chamber views, TTE allows for the measurement of CST size and diameter using M-mode [56]. However, the posterior location of the CS often results in suboptimal visualization with reduced sensitivity for small thrombi, especially in patients with obesity or lung disease [29, 57]. This limitation can lead to misinterpretations, such as mistaking CST for a left atrial mass or a coronary sinus hematoma for a thrombus [58, 59]. In contrast, TEE offers superior visualization of the CS and other posterior heart structures due to the proximity of the esophagus to the heart. TEE can more precisely visualize CST in the modified midesophageal 4-chamber view and the bicaval view than TTE [52, 60]. However, Echocardiography is limited in distinguishing thrombi from tumors due to insufficient tissue characterization [40, 52, 61, 62]. Ultimately, the decision between TTE and TEE depends on the clinical context, patient condition, and the availability of resources.

Computed Tomography (CT): CT is another valuable diagnostic tool for CST. It provides high-resolution images of the CS and surrounding structures, allowing for detailed visualization of thrombus and associated venous abnormalities. Non-contrast CT can provide valuable initial insights, particularly in identifying indirect signs of CST, such as dilated CS [33]. Since non-contrast CT lacks the resolution to clearly distinguish surrounding tissues, it can only reveal chronic organized thrombi as fluid-density dark areas within the CS [17], rather than acute thrombi. Contrast-enhanced CT (CECT) provides much greater diagnostic value for detecting CST. The use of intravenous contrast enhances the visibility of the CS and surrounding vasculature, making it possible to directly detect the thrombus and assess its size, location, and extent. Additionally, CECT is valuable for pinpointing venous obstructions or embolisms and provides detailed information about adjacent structures, helping identify underlying causes of CST [33]. Cardiac CT, a specific type of contrast-enhanced imaging, offers the added advantage of visualizing the coronary arteries and intracardiac structures simultaneously, which capability makes cardiac CT particularly suitable for CST evaluation [18, 63]. Overall, while non-contrast CT can serve as a useful first step in evaluating CST, CECT is the preferred imaging modality due to its ability to offer a more precise and comprehensive assessment.

Magnetic Resonance Imaging (MRI): MRI, particularly with contrast, can provide detailed soft-tissue characterization and is helpful in identifying CST [50, 57]. It also aids in assessing CS anatomy and identifying any congenital anomalies [64–66]. MRI is typically used when CT doesn’t provide sufficient diagnostic clarity. While offering precise thrombosis visualization, it is generally reserved for non-acute or complex cases.

Venography: Venography is typically used for complex cardiac or venous anatomical assessments, particularly in procedures requiring catheter or electrode placement [67, 68]. It is highly effective for diagnosing CST by providing clear and precise imaging of the CS and its branches. It can accurately determine the location, size, and extent of the thrombus [55], and is also useful for identifying other complex venous anomalies. However, venography is invasive and carries risks, such as thrombosis or venous injury. Thus, it is usually reserved for cases where non-invasive imaging is insufficient or detailed venous assessment is required before surgery.

## CST treatment strategies

Without standardized guidelines, CST treatment remains largely empirical. with anticoagulation guidelines providing some reference [69]. Based on previous research cases (Table 1), common options primarily include anticoagulation and surgical thrombectomy.

### Anticoagulation therapy

Anticoagulation is a cornerstone in managing CST, aimed at preventing thrombus extension and promoting clot resolution [57]. It can also serve as essential bridging therapy following surgical thrombectomy to prevent re-thrombosis during the postoperative period. Standard anticoagulation regimens typically involve the use of heparin for acute management, followed by long-term oral anticoagulation with warfarin or direct oral anticoagulants (DOACs), such as rivaroxaban or apixaban. The choice of anticoagulant and its dosing can be influenced by various patient-specific factors, which may impact the effectiveness of the treatment [1, 18].

Intravenous heparin administration should be emphasized in CST management [43, 44], as studies report cases where heparin led to thrombus resolution within a week [44]. In many instances, heparin quickly restores partial blood flow in the obstructed CS [41], improving circulation and often achieving full thrombus resolution within a month [43, 57]. This underscores heparin’s efficacy as a critical early treatment component for CST. However, Dabbah S documented a case where thrombosis recurred despite combined heparin and warfarin therapy, with an INR above 2.0^29^. This highlights a challenge in CST management, as some patients may experience re-thrombosis even within the therapeutic range. For such high-risk cases, an adjusted or intensified anticoagulation approach, including expand anticoagulation standards or consider combination therapy with additional anticoagulant agents to provide a higher level of protection. Close monitoring and supplementary strategies could further support vessel patency and help reduce the risk of re-thrombosis.

Novel anticoagulants, such as rivaroxaban and apixaban, are increasingly being recommended for the prevention and treatment of CST [49]. Compared to traditional anticoagulants, these newer agents offer simplified dosing and require less frequent coagulation monitoring, which can improve patient adherence [18, 52, 70, 71]. By directly targeting the coagulation cascade, they show potential in preventing CST formation and limiting thrombus progression. Although clinical experience and research data remain limited, the application of novel anticoagulants in managing CST presents an encouraging avenue for further investigation.

### Surgical thrombectomy

While anticoagulation remains the primary treatment for CST, surgical intervention becomes necessary in complex cases, particularly in acute scenarios where rapid symptom relief and hemodynamic stabilization are critical. Thrombectomy can provide immediate relief from obstruction, restoring blood flow and significantly improving cardiac function, effectively halting the progression of potentially fatal complications. Multiple cases document marked functional recovery after surgery [23, 30]. In large CSTs, even in asymptomatic patients, surgical intervention may be advisable, as illustrated by Wang Wei’s report of a 9 cm thrombus, where surgery was favored due to anticoagulation uncertainty and high risk of sudden complications [72]. Infected thrombi introduce additional complexity, where direct surgical removal not only alleviates the thrombus burden but also minimizes the risk of further inflammation and infection progression [38, 58]. particularly in cases where pharmacologic management alone proves insufficient [37, 38]. Additionally, imaging limitations can obscure mass identification within the CS, making surgery a definitive solution, as in Yamaguchi M’s case where a hemangioma was confirmed postoperatively [62]. When CST affects valvular function or involves congenital anomalies, surgery offers substantial benefits. In mitral or tricuspid valve abnormalities, backflow and venous stasis foster thrombosis, while surgical removal restores circulation and prevents hemodynamic decline [20, 23, 31, 40]. For congenital conditions [58, 67], which predispose patients to CST through altered hemodynamics, corrective surgery addresses both the structural anomaly and reduces CST incidence, enhancing long-term outcomes.

The presence of PLSVC complicates surgical planning. Thorough preoperative imaging and planning are crucial to address these anatomical variations. In cases where PLSVC is accompanied by CSA or an absent right superior vena cava, the PLSVC may serve as the primary retrograde drainage pathway, making ligation potentially life-threatening [11, 33]. If PLSVC coexists with URCS or connects to the left atrium, vascular reconstruction may become necessary. Careful preoperative assessment and strategy formulation are even more critical when PLSVC is associated with other cardiac anomalies, such as atrial septal defect, bicuspid aortic valve or coarctation of aorta [73, 74]. These situations highlight the importance of precise anatomical assessment and a carefully tailored surgical strategy to prevent severe complications.

The surgical options for managing CST can be categorized based on invasiveness and specific patient needs. Open surgical thrombectomy involves direct access to the CS, typically under cardiopulmonary bypass, and may require a thoracotomy. This approach is suited for cases with acute cardiac dysfunction [23, 40], complete obstruction [23, 30, 63], complex structural abnormalities [20, 58, 62], infections [37], or large thrombi [72], allowing for thorough thrombus removal and correction of affected adjacent valves or vascular abnormalities. However, open surgery demands greater physical resilience and carries higher risks and recovery burdens, making it less suitable for high-risk patients [33, 75]. Minimally Invasive or Catheter-Assisted Thrombectomy uses catheters to access the CS. with techniques like aspiration thrombectomy (suctioning the clot) [3, 4]. angioplasty [68], and mechanical thrombectomy devices to break up and remove the thrombus. This approach is less invasive, usually associated with quicker recovery, and often performed under local anesthesia, making it suitable for partial obstructions, smaller thrombi, or patients at higher surgical risk. However, catheter-based techniques may not be as effective for large or organized complex thrombi, sometimes requiring additional procedures if complete removal isn’t achieved. Endovascular stenting may be used to maintain CS patency in cases at risk of recurrent thrombosis, such as persistent hypercoagulable states [76]. Additionally, when the nature of the lesion (e.g., thrombus versus tumor) cannot be definitively determined, or when complete removal of the thrombus or mass is not feasible without causing damage to the CS wall, endovascular stenting serves as a viable alternative. This approach provides long-term vessel patency following thrombectomy and is generally considered a secondary measure, with at least one case report documenting its successful application in preventing recurrent CST [40]. However, its indications require further validation through additional case studies.

In addition, postoperative anticoagulation is often necessary to prevent recurrence and maintain CS patency [4, 40, 58]. Combining surgery with anticoagulation offers a comprehensive strategy, providing both immediate thrombus resolution and long-term prevention, particularly beneficial in high-risk or complex cases.

### Post-Follow-Up

A structured post-treatment follow-up is essential for monitoring recurrence and managing complications in patients treated for CST. TTE offers a convenient and effective method for routine follow-up and is widely recommended for this purpose. However, its inherent limitations make it insufficient as the sole modality for precise postoperative monitoring. TEE serves as a valuable complement to TTE by providing superior imaging, but its requirement for sedation or anesthesia limits the practicality in routine follow-up. High-resolution CT provides thin-slice scanning, allowing for more precise imaging and making it a powerful approach for detecting CST recurrence and screening for subtle changes in thrombus size or location. MRI complements CT effectively, especially in cases requiring soft-tissue characterization or when CT findings are inconclusive. Nevertheless, it is unsuitable for uncooperative patients and necessitates confirmation of stent material compatibility to ensure both safety and imaging accuracy. While venography delivers the most detailed visualization, its invasive nature and associated risks limit its use in routine follow-up.

In cases where anticoagulation therapy is part of the post-treatment regimen, routine blood tests to monitor coagulation parameters and adjust dosing are important to maintain therapeutic efficacy and minimize bleeding risks. For patients with uncorrected underlying risk factors, such as certain diseases or structural abnormalities, follow-up frequency should be increased to ensure timely detection and management of potential complications.

## Conclusion

CST is a rare but potentially life-threatening condition that presents significant diagnostic and therapeutic challenges. It often manifests with nonspecific symptoms and can progress rapidly to severe complications if not promptly recognized and managed. Anticoagulation remains the cornerstone of treatment, while surgical intervention expands treatment options, especially for acute and complex cases. Deep recognizing and understanding are essential for guiding interventions and improving outcomes. Multidisciplinary approaches will be valuable in refining treatment protocols. Given the high mortality rate associated with CST, it should always be considered as a differential diagnosis whenever relevant symptoms arise.

## Methodology of Literature Review

This review systematically explored literature on CST. Keywords like “coronary sinus thrombosis” and “coronary sinus imaging” were used to search databases including PubMed, Web of Science, Scopus, and Google Scholar for publications from January 1974 to December 2024. Inclusion criteria included studies addressing CST, with or without PLSVC, that provided diagnostic imaging, clinical presentation, or management and were published in English (An exception was made for a Japanese article due to its unique and valuable clinical insights [34]). Exclusion criteria included studies lacking sufficient clinical or imaging details, non-thrombotic coronary sinus masses, non-coronary sinus cardiac thrombi, and inaccessible publications.

## Data Availability

No datasets were generated or analysed during the current study.
